# Cross-border simulation training for German and Polish emergency medical teams is feasible: conception and evaluation of a bilingual simulation training

**DOI:** 10.1186/s12909-023-04823-y

**Published:** 2023-11-13

**Authors:** Marie-Luise Ruebsam, Dorota Orsson, Bibiana Metelmann, Jakub Orsson, Klaus Hahnenkamp, Camilla Metelmann

**Affiliations:** Department of Anaesthesia, Intensive Care, Emergency and Pain Medicine, University Medicine of Greifswald, Ferdinand-Sauerbruch-Straße, 17475 Greifswald, Germany

**Keywords:** Simulation, Cross-border, Emergency medicine, Bilingual, Team training

## Abstract

**Background:**

Cross-border cooperation of emergency medical services, institutions and hospitals helps to reduce negative impact of national borders and consecutive discrimination of persons living and working in border regions. This study aims to explore the feasibility and effectiveness of a cross-border bilingual simulation training for emergency medical services within an INTERREG-VA-funded project.

**Methods:**

Five days of simulation training for German and Polish paramedics in mixed groups were planned. Effectiveness of training and main learning objectives were evaluated as pre-post-comparisons and self-assessment by participants.

**Results:**

Due to COVID-19 pandemic, only three of nine training modules with *n* = 16 participants could be realised. Cross-border-simulation training was ranked more positively and was perceived as more useful after the training compared to pretraining. Primary survey has been performed using ABCDE scheme in 18 of 21 scenarios, whereas schemes to obtain medical history have been applied incompletely. However, participants stated to be able to communicate with patients and relatives in 10 of 21 scenarios.

**Conclusion:**

This study demonstrates feasibility of a bilingual cross-border simulation training for German and Polish rescue teams. Further research is highly needed to evaluate communication processes and intra-team interaction during bilingual simulation training and in cross-border emergency medical services rescue operations.

## Background

Patients experiencing medical emergencies have to be treated as fast as possible, regardless of their current localisation [1]. Borders have been identified to increase the time it takes the emergency medical service (EMS) to reach the patient and to provide in-hospital treatment. Frequently, the closest EMS provider is located in the neighbouring country. Therefore, borders may result in higher costs, lower quality of life and higher mortality. Rejecting cross-border assistance can be considered morally disputable, because cross-border (CB) cooperation of EMS, institutions and hospitals helps to reduce the discrimination of people living in border regions [2, 3].

Cooperation among emergency medicine providers is based on legal agreements between European countries and regulated by regional cooperation treaties. However, cooperation requires a particularly high degree of coordination and regulation since the EMS is organised differently throughout Europe [4]. Along the German borders, level of cross-border cooperation differs due to e.g. language barriers, political development and degree of regional networks and activities. CB collaboration at the Polish-German border is still at the beginning. Based on the German-Polish framework contract, a cooperation treaty has been signed in 2020 between the Voivodship Westpomerania (Republic of Poland) and the County of Vorpommern-Greifswald (Federal Republic of Germany) [5]. It defines operational areas, means of notifying the neighbour dispatch centre, transfer of patients, financial aspects, documentation, and quality management.

With this increasing degree of cooperation medical and linguistic challenges arise simultaneously. Diagnostics and treatment of emergency medicine patients need to be focused and prioritised. Relevant skills can’t be trained “on the job” only, due to the vulnerability of critical ill patients including a low margin of error, timely completion of critical procedures and the need of structured feedback [6]. Although simulation has been identified as an adequate method in medical education [7], there is only limited knowledge about its integration and effectiveness in CB-EMS rescue operation training. Beuken et al. showed a strong desire of paramedics working in CB situations to get to know the professionals from the neighbour countries and to train together. Skill trainings in interprofessional, intercultural settings create a shared understanding of CB collaboration [8]. Providing health care requires a culturally and linguistically appropriate approach impacting acceptance, satisfaction of patients and quality of care [9, 10]. A systemic study analyzing barriers of cross-border EMS identified bilingual communicational competencies of EMS employees and dispatchers as essential [4].

This study aims to explore feasibility, effectiveness, and challenges of a cross-border bilingual simulation training for emergency medical teams.

## Methods

### Study population and simulation facility

Within the scope of the INTERREG-VA-funded project “InGRiP—Integrated cross-border medical services Pomerania/Brandenburg”, a language and simulation training for German and Polish paramedics along the border was conducted [11]. A discontinuous three-week language module in separate German and Polish groups was followed by a refresher course. Language training courses taught EMS specific vocabulary as well as grammar and syntax [12].

The following simulation training was designed for small mixed groups with an 1:1 ratio of German and Polish paramedics on five consecutive days. 82 paramedics of ground ambulance services in the border region (Vorpommern-Greifswald, Märkisch-Oderland, and Voivodship Westpomerania), and air ambulance services (DRF Stiftung Luftrettung and Lotnicze Pogotowie Ratunkowe) were scheduled to participate.

The composition of the mixed German and Polish teams taking part in the simulation scenarios was carried out by both the linguist and the emergency medical instructors. Professional experience and communicational competencies prior to simulation training were considered.

### Conception and realization

Conception was based on three learning objective categories: (a) proper and barrier-free communication during CB-EMS rescue operations, (b) emergency medical knowledge and (c) specific medico-legal aspects of CB-EMS between Germany and Poland.

These learning objectives were based on factors, which limit CB collaboration in emergency medicine, such as legal insecurities, communication barriers, and negotiation of political priorities [2].

13 bilingual scenarios covering a broad range of typical pre-hospital emergencies were developed by a German and Polish speaking project team member. Simulation scenarios and following bilingual debriefing were led by an interprofessional team of a qualified EMS instructor supported by a bilingual course coordinator. Simulation training took place in the simulation facility of the Polish Ground Ambulance Services in Misdroy, Poland.

### Outcome measurements and evaluation

In order to explore feasibility, effectiveness, and challenges of a cross-border bilingual simulation training program for emergency medical teams, we aimed to measure (1) effectiveness based on the first two levels of the Kirkpatrick model (2) team performance, and (3) realization of learning objectives using a triangulation approach. To achieve this, a questionnaire in a pre-post-design was combined with additional questionnaires after each scenario. Table 1 shows an overview of the questionnaires used. These will be explained in the following paragraphs.

**Table 1:** Overview of questionnaires

Pre-post-design	After each scenario
“training evaluation inventory” by Ritzmann [13]	Learning objectives (11 items)
Transfer of training by Baldwin, Ford and Kramer [14, 15]	Team Emergency Assessment Measure (TEAM)’ questionnaire by Cooper [16]
Knowledge test regarding learning objectives	

Effectiveness of training can be determined using the Kirkpatrick model which consists of four levels validated to assess the value and effectiveness of a training program or a learning intervention [17–20]. To evaluate the effects of simulation training on affective reaction, expectations, knowledge, and motivation towards training, participants were asked to complete a ‘pre-training-survey’ before attending the first simulation training and a ‘post-training-survey’ after completing all training sessions. The “training evaluation inventory” by Ritzmann provided validated questions to the training outcome dimensions of Kirkpatrick level 1 “reaction” (such as “subjective enjoyment”, “perceived usefulness”, “perceived difficulty”), and level 2 “learning” (such as “subjective knowledge gain” and “attitude towards training”) [17]. As motivation to transfer and to apply knowledge in daily practice are important outcomes, the process model developed by Baldwin and Ford was integrated and questions validated by Kramer et al. were included [18, 19]. Corresponding questions to these categories were transferred to context. A symmetrical 5-point Likert-scale from 0 [strongly disapprove] to 4 [strongly approve] was used. Furthermore, a knowledge test assessing participants` language skills, emergency medical knowledge, and CB-EMS knowledge, was added to the pre-post questionnaires to get an impartial measurement of the three learning objectives. The pre-training-survey additionally contained eight questions concerning participants’ characteristics.

After each scenario, participants were asked to complete a short questionnaire consisting of 11 questions regarding defined learning objectives. These questions could be answered on a 3-point Likert scale. To assess non-technical skills, an adapted ‘Team Emergency Assessment Measure (TEAM)’ questionnaire with 11 items validated for resuscitation scenarios was used [20].

Evaluation of team performance and realization of learning objective during each scenario was planned as comparison of self-perception of participants and external observation, as the former may be confounded by emotion and social desirableness [21]. Therefore, an external observer was to complete the same questionnaires. As this training was bilingual and participants were not fluent in the neighbouring language, questionnaires were provided in German and Polish. The underlying questions and questionnaires were originally designed in English or German and validated for those languages. Thus, each question was translated into Polish through a multi-stage process by our linguist. This process involved adaption of intercultural differences, forward and backward translation, and an internal test of intelligibility and consistency of questions.

### Statistical methods

Survey software EvaSys® (Electric Paper Evaluationssysteme GmbH, Lueneburg, Germany) was used and data set was analysed using SPSS® (Statistical Package for the Social Sciences, IBM, Armonk, New York, USA).

Participants’ characteristics were assessed descriptively. For pre- and post-training evaluation, questions of respective categories were summarised. Results are presented as means ± standard deviation (SD). Comparison of means was planned using paired t-test for normal distributed data and Wilcoxon-test for not normal distributed data. If questions were not or not unambiguously answered, these values were excluded from analysis.

A correlation of self and external perception of team performance was planned to provide information on the quality of the self-assessment of emergency paramedics during simulation training. Questionnaires were anonymised to maintain confidentiality and informed consent has been obtained from all participants previously to simulation training.

## Results

### Feasibility: Impact of COVID-19 pandemic on realization

To ensure safety of participants and to adhere to national legal regulations during COVID-19 pandemic, the number of people attending simulation training had to be reduced rigorously. Consecutively, no external observation or on-site support to distribute and collect questionnaires after each simulation scenario was possible. With increasing COVID-19 incidences in March 2020, the integrated CB training had to be stopped after three out of nine training modules. In October 2020, training was restarted in digital hybrid form. However, no integrated CB on-site training was feasible in the remaining term of project, which ended in February 2021. The resulting small number of completed questionnaires only enabled descriptive presentation of data from pre- and post-training survey, as well as the team emergency assessment measure and evaluation of CB-EMS specific aspects.

### Characteristics of participants

A total of 16 paramedics from Germany and Poland participated in three one-week simulation training sessions and 13 paramedics (81%) completed the pre-training-survey. Of these 13 participants, 12 were male and one female; 6 from Germany, 7 from Poland. Age ranged from 24 to 48 years (mean 37.5 years) and participants had 2 to 27 years of professional experience (mean 14.2 years). Three German participants stated to be Polish native speakers.

### Pre- and post-training evaluation

**Fig. 1:**
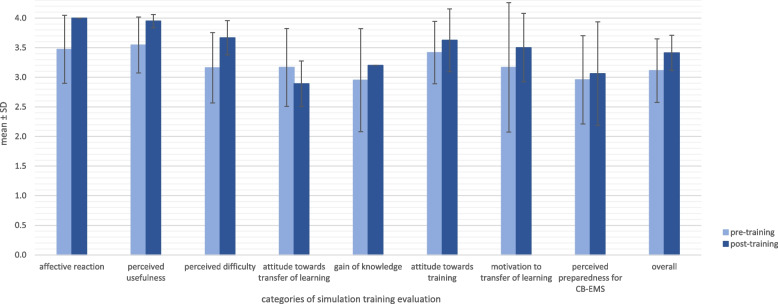
Pre- and post-training comparison of simulation training categories as means ± standard deviation

Overall expectations of training were high (3.1 ± 0.5) (Fig. 1). Highest scored elements were “perceived usefulness” (3.5 ± 0.5) and “positive attitude towards training” (3.5 ± 0.6). Participants were less optimistic and rather indecisive about expected gain of knowledge (3.0 ± 0.9). They were unsure about their preparedness for CB-EMS (3.0 ± 0.7). In post-training-survey, response rate has been considerably lower (n = 5; 31%) compared to pre-training evaluation (n = 13; 81%). After attending CB simulation training, it was ranked more positively (pre 3.1 ± 0.5 vs post 3.4 ± 0.3).

Perceived usefulness of training (3.5 ± 0.6 to 4.0 ± 0.0) and affective reaction towards training increased (3.5 ± 0.6 to 4.0 ± 0.1). Perceived difficulty was scored higher after training (3.2 ± 0.6 vs 3.7 ± 0.3). Perceived difficulty implies duration of simulation training (2.7 ± 1.1 vs 3.3 ± 1.0), increased understandability of content (3.5 ± 0.5 vs 4.0 ± 0.0) but also a considerable increase regarding the ability to communicate with instructors and participants during training (2.8 ± 0.7 vs 3.8 ± 0.5) and ability to follow simulation scenarios thematically (3.1 ± 0.9 vs 4.0 ± 0.0).

Before simulation, the participants ranked transferability of training content higher than afterwards (3.2 ± 0.7 vs 2.9 ± 0.4). In this category, participants were asked to score requirements and realism of scenarios compared to daily CB-EMS practice. Both did not differ before and after training (3.3 ± 1.0 vs 3.3 ± 1.2 and 3.3 ± 0.7 vs 3.0 ± 0.0). Participants assumed that daily CB-EMS operations will not be as challenging as during simulation (3.1 ± 1.1 vs 2.3 ± 1.2).

There was only a small increase (3.0 ± 0.7 vs 3.1 ± 0.9) regarding preparedness for CB-EMS operations.

Comparing correct answers of pre- and post-training knowledge test, high levels of language skills could be observed (94% compared to 95%), whereas, participant`s emergency medical knowledge (60% vs 72%) and CB-EMS knowledge (2% vs 40%) started low but improved.

### Self-assessment of learning objectives

**Fig. 2:**
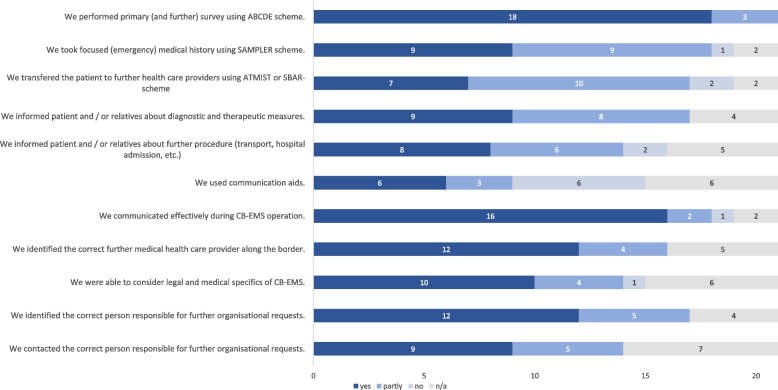
Evaluation of learning objectives by self-assessment (n = 21) with absolute number of answers

A total of 23 post-scenario-questionnaires were completed. Out of these two questionnaires on learning objectives had to be excluded from analysis because of inconsistent answers (Fig. 2). Primary survey using ABCDE scheme has been performed in 18 out of 21 scenarios (item 1). Schemes like SAMPLER to explore emergency relevant case history and ATMIST/SBAR to transfer the patient to further health care providers (item 2 and 3) have been applied partly. Participants were able to communicate with patients and relatives concerning diagnostic and therapeutic procedures in more than half of the scenarios (item 4 and 5). Mostly, no communication aids were used (item 6). Identification of appropriate contact person and organising of subsequent treatment was done in most scenarios (item 7–11).

### Self-assessment of team performance

All 23 post-scenario-questionnaires could be included in the analysis. Mean TEAM scores were clustered at high-range (3.5 ± 0.1). The statement “team morale was positive” received highest rating (mean: 3.7 ± 0.6), whereas the corresponding statement concerning team climate (“the team acted with composure and control”) received lowest rating (3.2 ± 1.0) (Table 2).

**Table 2:** Results of ‘Team Emergency Assessment Measure (TEAM)’ questionnaire presented as mean and standard deviation (SD)

Category	Number of valid answers (n)	Mean ± SD
**Leadership**		
Direction	16	3.4 ± 1.4
Global Perspective	19	3.4 ± 0.8
**Teamwork**		
Communication	20	3.4 ± 0.7
Co-Operation and Co-Ordination	19	3.6 ± 0.6
Team morale	20	3.2 ± 1.0
	19	3.7 ± 0.6
Adaptability	19	3.6 ± 0.5
Situation Awareness (Perception)	20	3.6 ± 0.5
Situation Awareness (Projection)	18	3.5 ± 0.6
**Task Management**		
Prioritisation	19	3.6 ± 0.6
Clinical Standards	18	3.4 ± 0.8
**Global Rating**	20	8.2 ± 2.0
**TEAM Score overall**	13	39 ± 5.3

## Discussion

### Results in short

CB-EMS simulation training is feasible and positively reviewed by German and Polish paramedics. Participants stated that they used ABCDE-scheme for assessment and treatment. Furthermore, participants assessed, that in most scenarios they were able to communicate tasks, diagnosis and treatment to colleagues, patients, and relatives in the neighbour's language.

## Discussion

### Feasibility of cross-border bilingual simulation training

Three one-week simulation training sessions with a total of 16 participants could be realised. Although the COVID-19 pandemic led to an earlier termination of the study protocol, we could still show, that a cross-border simulation training for German and Polish emergency medical teams is feasible.

### Effectiveness: Pre- and post-training evaluation

Participants stated that scenarios were too challenging compared to their CB-EMS reality. This might be since simulation is currently no standard method in EMS post-graduate education. Furthermore, simulation scenarios might have been too complex as they did not only cover a broad range of emergencies in critical ill adults and children but included the necessity to perform invasive technics such as thoracic decompression or advanced airway management.

Participants were able to improve their emergency medicine related knowledge by 20% and knowledge regarding medico-legal aspects of CB-EMS by about 100% comparing pre- and post-test results. In contrast to these findings, scores indicate that training did not enhance perceived preparedness of participants for CB-EMS operations.

### Effectiveness: Self-assessment of learning objectives

Regarding self-assessment of learning-objectives, it is important to notice, that self-assessment is no reliable predictor of effectiveness of training, gain of knowledge and skills. Participants tend to overestimate their performance [22]. Furthermore, overall positive expectations towards simulation training and self-assessment of within-training performance could be caused by response bias or voluntarily participation.

Participants ranked overall CB-EMS operation as “communicatively effective”. Intra-team cooperation, coordination, prioritisation, and communication were rated as highly positive. However, participants indicated that they only used ABCDE scheme and no communication aids. Participants perceived improvement in language skills during the simulation training as low. These findings may prompt the hypothesis that even when confronted with language barriers the use of international acknowledged schemes such as ABCDE facilitate mixed EMS teams to act timely, cooperatively, and effectively. As most of the procedures demand direct communication with the patient, knowledge of patient's language or access to communication tools is mandatory to provide safe and high-quality medical aid and to achieve patient’s satisfaction [23]. Acquisition of specialised language mandatory for CB-EMS can be improved through tandem training programme consisting of simultaneous learning and using the foreign language. From the linguistic point of view, verification of communication process is an essential task during further CB German-Polish simulation trainings [12, 24].

### Effectiveness: Self-assessment of team performance

According to Cooper et al., an overall TEAM score of 34 and a global rating of 8.2 indicate a good team performance [16]. Participants of our study rated their overall TEAM score with 39 ± 5.3 and reached a global rating of 8.2 ± 2.0. The participating paramedics experienced the bilingual teamwork as supportive and collaborative.

### Challenges of cross-border bilingual simulation training

CB-EMS operations only affect a small part of EMS in Germany and Poland. Currently, each district in Germany has to sign an own cooperation contract with its Polish neighbour district. As these cooperation contracts are very specific for the particular region, no transfer of standardised knowledge among different EMS-areas is possible resulting in the necessity to train paramedics and emergency physicians to act in congruence to existing law within a CB-EMS operation. Schwarzenberg showed that paramedics, who routinely work in CB-EMS still have questions regarding legal issues [2]. Simulation training can be considered as successful and suitable, but further training is highly necessary to improve quality and patient safety in CB-EMS.

### Limitations of the study

As the number of participants was determined within the framework of the project based on practical-oriented requirements, this planning cannot be considered as a calculation of an a priori sample size and therefore may limit the validity of our results.

Due to restrictions caused by COVID-19 pandemic, only a fifth of the participants initially scheduled for training were able to take part. As three participants withdrew their permission to evaluation and five participants did not fill out the questionnaire at all or only incompletely, the resulting overall dropout rate of 62% (n = 8) limits external validity. Furthermore, limited data did only allow for descriptive presentation and trending. The comparatively high response rate prior training (81%) might be because this questionnaire was distributed during introduction presentation by the medical head of faculty [25]. Drop out response rates might be explained by lack of reminders to answer questionnaires after each scenario because instructors were either involved in debriefing or preparing the next scenario. Another reason could be length and complexity of questionnaires causing increasing fatigue [26].

Due to COVID-19 pandemic, this evaluation lacks external (peer) observation. This may affect value and significance of results as several studies showed only weak or no associations between physician’s self-assessment and external rating [22]. When comparing a priori and posteriori self-assessment of students with their actual performance in oral examination, students failed to self-assess their performance successfully and tended to be more optimistic [27]. Students tended to particularly overestimate their communicative skills in contrast to medical knowledge [28]. Reasons for this inaccuracy can include a different frame of reference of the assessor vs self and measurement error [29].

As the simulation training was conducted in Polish and German, the questionnaire was translated. Though this translation process involved an internal test of intelligibility and consistency, an external study of validity of translation was not performed.

A mayor limitation is that effects of training could not be assessed on a higher level [17]. This involves potential behavioural changes caused by simulation training and influenced by the stated motivation of learning transfer as well as the direct impact on daily practice of the paramedics and therefore quality of care provided in CB-EMS.

## Conclusions

This study demonstrated feasibility of bilingual CB simulation training for German and Polish rescue teams. Although participants expected higher increase in language-skills, they stated that in most scenarios they were able to communicate adequately with colleagues, patients, and relatives in the neighbour's language. In future CB-simulation training, it may be necessary to reduce complexity of learning objectives regarding emergency medical knowledge and skills in order to focus on CB-communication.

Therefore, further research is highly needed to evaluate communication processes during bilingual simulation training and intra-team interaction in CB-EMS rescue operations.

## Data Availability

The datasets used and analysed during the current study are available from the corresponding author on reasonable request.

## Abbreviations

CB: Cross-border
EMS: Emergency medicine service
SD: Standard deviation
TEAM: Team Emergency Assessment Measure
